# Association between End-Stage Renal Disease and Incident Diabetes Mellitus—A Nationwide Population-Based Cohort Study

**DOI:** 10.3390/jcm7100343

**Published:** 2018-10-11

**Authors:** Pin-Pin Wu, Chew-Teng Kor, Ming-Chia Hsieh, Yao-Peng Hsieh

**Affiliations:** 1 Department of Internal Medicine, Changhua Christian Hospital, Changhua 500, Taiwan; 125630@cch.org.tw (P.-P.W.); 179297@cch.org.tw (C.-T.K.); 146446@cch.org.tw (M.-C.H.); 2 School of Medicine, Kaohsiung Medical University, Kaohsiung 80708, Taiwan; 3 School of Medicine, Chung Shan Medical University, Taichung 40201, Taiwan

**Keywords:** burnt-out diabetes, chronic kidney disease (CKD), dialysis, end-stage renal disease (ESRD), incident diabetes mellitus (DM), insulin resistance

## Abstract

Background: Glucose is one of the constituents in hemodialysates and peritoneal dialysates. How the dialysis associates with the incident diabetes mellitus (DM) remains to be assessed. Methods: The claim data of end-stage renal disease (ESRD) patients who initiated dialysis from and a cohort of matched non-dialysis individuals from 2000 to 2013 were retrieved from the Taiwan National Health Insurance Research Database to examine the risk of incident DM among patients on hemodialysis (HD) and peritoneal dialysis (PD). Predictors of incident DM were determined for HD and PD patients using Fine and Gray models to treat death as a competing event, respectively. Results: A total of 2228 patients on dialysis (2092 HD and 136 PD) and 8912 non-dialysis individuals were the study population. The PD and HD patients had 12 and 97 new-onset of DM (incidence rates of 15.98 and 8.69 per 1000 patient-years, respectively), while the comparison cohort had 869 DM events with the incidence rate of 15.88 per 1000 patient-years. The multivariable-adjusted Cox models of Fine and Gray method showed that the dialysis cohort was associated with an adjusted hazard ratio (HR) of 0.49 (95% CI 0.39–0.61, *p* value < 0.0001) for incident DM compared with the comparison cohort. The adjusted HR of incident DM was 0.46 (95% CI 0.37–0.58, *p* value < 0.0001) for HD and 0.84 (95% CI 0.47–1.51, *p* value = 0.56) for PD. Conclusions: ESRD patients were associated with a lower risk of incident DM. HD was associated with a lower risk of incident DM, whereas PD was not.

## 1. Introduction

The increasing prevalence of chronic kidney disease (CKD) has a global impact on healthcare management and socioeconomic systems worldwide. Similarly, the prevalence of diabetes mellitus has also been trended high in the general population, especially in the obese and aging [1]. Both of these two entities share many common cardiovascular morbidities and may influence each other in an enhancing manner.

Uremia occurs as a result of enormous retention of various substances when the kidney function is worsening progressively. The acceptable renal replacement therapy includes hemodialysis (HD), peritoneal dialysis (PD), and kidney transplant. Glucose is used as one of the constituents in hemodialysates and peritoneal dialysates [2,3]. Thus, one may expect the higher incidence rate of diabetes mellitus (DM) for dialysis patients owing to the more glucose uptake from the dialysate. However, the epidemiological data on the association of glucose load with incident DM have concluded contradictory results [4,5].

Insulin resistance (IR) is one of the key determinants in the development and progression of DM. IR has existed across all the CKD spectrums and gets exacerbated with the deterioration of renal function as uremia toxins contribute to IR [6,7]. Metabolic acidosis, even of a slight degree, can suppress insulin release and induce IR in CKD [8]. Once non-DM end-stage renal disease (ESRD) patients undergo dialysis treatment, the reduction of uremia toxin and alleviation of metabolic acidosis by dialysis may partially alleviate the degree of IR, thus mitigating the diabetic risk. DM is the leading cause of ESRD worldwide and about 40% of ESRD patients were attributed to diabetic nephropathy [9]. How the dialysis associates with the incident diabetes remained to be assessed. Taiwan has the highest prevalence and incidence rates of ESRD in the world. Therefore, we conducted a retrospective national cohort study using the Taiwan National Insurance Research Database (NHIRD) to compare the risk of incident DM between ESRD patients undergoing PD or HD and non-dialysis patients. In addition, we also determined the risk factors associated with incident DM.

## 2. Materials and Methods

### 2.1. Data Source and Study Population

This nationwide retrospective study was conducted using the data retrieved from the Longitudinal Health Insurance Database (LHID), which was randomly selected from the Taiwan National Health Insurance Research Database (NHIRD) and contained the entire claim data for one million beneficiaries. The Taiwan NHIRD was released by the Taiwan National Health Research Institute for scientific research. The Taiwan National Health Insurance (NHI) program has been launched since 1995 and all citizens are enrolled in the program, except prisoners, with a coverage rate of >99%. Therefore, The Taiwan NHIRD can represent the utilization conditions of medical resources for the 23 million residents and is one of the largest databases universally. The NHI adopted the International Classification of Diseases-9th revision, Clinical Modification (ICD-9-CM) for medical payments applications. Patients with ESRD who underwent dialysis treatments will be issued a related catastrophic illness card and the copayment was waived. The Bureau of NHI audits the computerized claim data for medical expenses regularly and those contracted institutions with improper charges or malpractice will face heavy penalties, thus ensuring accurate medical coding. Many high-quality researches have been published using the Taiwan NHIRD [10,11,12]. This study was exempted from informed consents because the personal identification data were encrypted and transformed in the NHIRD. The Institution Review Board of Changhua Christian Hospital reviewed and approved all the study proposals.

### 2.2. Study Design

This study was conducted using the inpatient and outpatient claim data from the LHID from 1996 to 2013. Patients who were at the age of 18–100 years and had started maintenance dialysis therapy for at least 90 days between the periods from 2000 to 2013 were enrolled in the dialysis cohort. The date of the first ESRD diagnosis was referred to as the index date. Patients who had undergone dialysis for ESRD from 1996 to 1999, received kidney transplant before the index date, type 2 DM before the index date or type 1 DM throughout the entire period, or incomplete demographics were excluded. Dialysis patients were further categorized as PD and HD groups according to their initial dialysis modality. The reference non-dialysis cohort was recruited from the same dataset with four controls matched to each one dialysis patient by age, gender, and the index year after excluding enrollees with CKD, ESRD, or renal transplant throughout the study period or DM before the index date. Follow-up data for the cohorts was reviewed from the index date until the date of incident DM, the end of 2013 or censored due to death, whichever occurred first.

### 2.3. Definition of ESRD, DM and Other Comorbidities

The inpatient and outpatient reimbursement data from LHID were linked to define the baseline demographic features and clinical conditions for both cohorts. Individuals were having any comorbidity if they fulfilled the following rules: One diagnostic code at discharge or at least two diagnostic codes in outpatient claims. To minimize the accidental miscoding in the outpatient reimbursement data, the diagnosis from outpatient encounters also required that the first and last diagnoses within one year were at least 30 days apart. ESRD on dialysis was ascertained from the catastrophic illness certificates with the code 585. DM was diagnosed only when there were both DM codes (ICD-9 code 250) and the use of anti-diabetic agents.

Aside from demographic data (e.g., sex, age, and residency area), we also collected information about comorbidities and drug treatments from the LHID. The baseline comorbidities included hypertension (ICD-9 codes 401–405), ischemic heart disease (ICD-9 codes 410–414), congestive heart failure (CHF) (ICD-9 code 428), cerebrovascular disease (ICD-9 codes 430–438), rheumatoid disease (ICD-9 codes 446.5, 710.0–710.4, 714.0–714.2, 714.8, 725.x), gout (ICD-9 code 274), and chronic obstructive pulmonary disease (ICD-9 codes 491, 492, 496). We also obtained information on pharmacotherapy regarding statins, anti-hypertensive drugs, analgesics, and glucocorticoids for multivariate adjustment.

## 3. Statistical Analysis

Summary descriptive data were shown as mean ± standard deviation (SD) and frequency with percentage for continuous and categorical covariates, respectively. The distributions of variables between the case cohort and matched reference cohort were compared using Student’s test, or Mann-Whitney test and the Chi-square test or Fisher’s exact test, as appropriate.

The outcome of interest in this study was incident DM event. The incidence rate was calculated as the number of new-onset DM cases divided by the follow-up time and expressed as the number of events per 1000 person-years for the dialysis and comparison groups. As death, a competing risk for the development of incident DM, was censored, we ran the Fine and Gray competing risk models with hazard ratio (HR) and 95% confidence interval (CI) to compare the DM risk between the cohorts. We also compared the risk of mortality (non-DM death), death without DM, between the cohorts. The association was further examined by stepwise cause-specific Cox proportional hazard models with SLENTRY = 0.15 and SLSTAY = 0.15. The HRs of incident DM for dialysis cohort versus comparison cohort were adjusted for all the baseline variables. The propensity score was calculated by logistic regression analysis and was used to adjust in the Cox regression model to reduce bias from unmeasured confounding factors. Four levels of sensitivity tests was performed as (1) adjusted for propensity score; (2) kidney transplant as a censored covariate; (3) change of renal replacement therapy as a time-dependent covariate; and (4) change of dialysis mode or kidney transplant as a censored event. The associations were also assessed in subgroups stratified by gender, age, income, and the number of medical visits in one year after study entry. Risk factors for incident DM were determined in the entire study population, comparison cohort and ESRD dialysis cohort, respectively. Statistical analyses were done using IBM SPSS Statistics for Windows, Version 22.0 (IBM Corp., Armonk, NY, USA). A two-sided *p*-value was set to < 0.05 with statistical significance.

## 4. Results

### 4.1. Patients’ Baseline Characteristics

**Figure 1:**
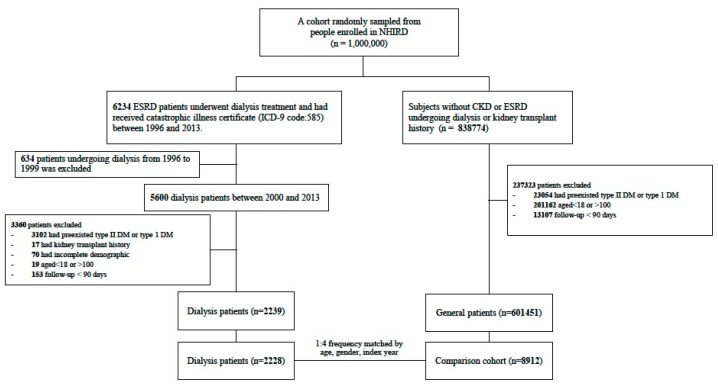
Flowchart of patient selection processes for the end-stage renal disease (ESRD) cohort and the comparison cohort.

The enrollment flowchart was depicted in Figure 1, demonstrating that after the matching processes a total of 2228 dialysis cases (2092 HD; 136 PD) and 8912 control cases were recruited from 2000 to 2013 for the analysis. The differences of the baseline patient characteristics between the dialysis and comparison cohorts were compared in the Table 1. As expected, the ESRD dialysis cohort had more medical visits, higher prevalence of most comorbidities, and higher proportion of patients taking medication than the comparison cohort.

### 4.2. Prevalence of Incident DM and Mortality Rate in the Dialysis and Comparison Cohorts

**Figure 2:**
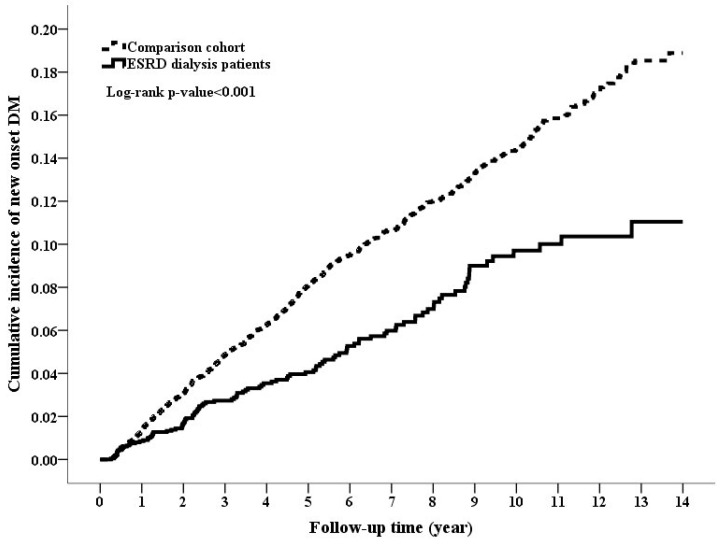
Cumulative incidence rate of diabetes mellitus between the end-stage renal disease (ESRD) cohort and the comparison cohort (*p*-value < 0.001, Log-rank test).

**Figure 3:**
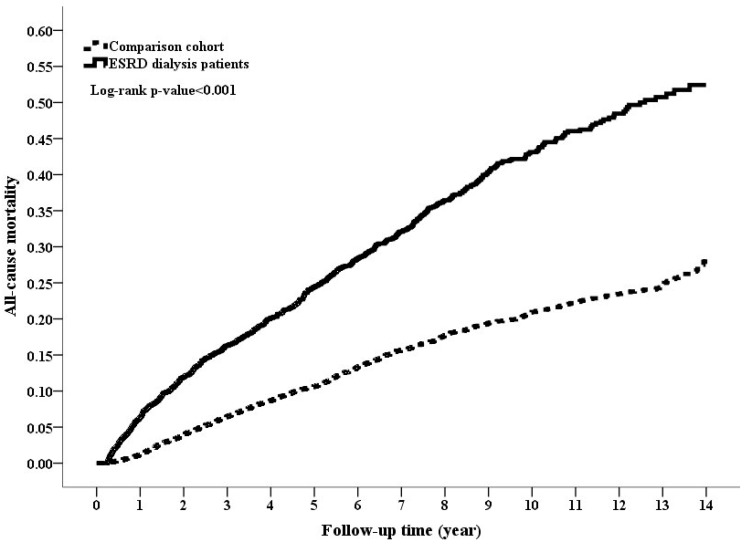
Cumulative incidence rate of mortality between the end-stage renal disease (ESRD) cohort and the comparison cohort (*p*-value < 0.001, Log-rank test).

During the follow-up period of around five–six years, the dialysis cohort had lower rate of DM and higher death rate compared to the comparison cohort (4.89% vs. 9.75%, *p* value < 0.001 for DM; 30.12% vs. 14.03% for death, *p* value < 0.001, Table 1). The cumulative incidence rate of DM was significantly lower in the dialysis cohort than in the comparison cohort, while the mortality rate was higher in the dialysis cohort than in the comparison cohort (Figure 2 and Figure 3; log-rank test, *p* value < 0.001 and *p* value < 0.001, respectively). The PD and HD patients had 12 and 97 DM events (incidence rates of 15.98 and 8.69 per 1000 patient-years, respectively), while the comparison cohort had 869 DM events with the incidence rate of 15.88 per 1000 patient-years (Table 2). The multivariable-adjusted Cox models of Fine and Gray method showed that dialysis cohort was associated with an adjusted HR of 0.49 (95% CI 0.39–0.61, *p* value < 0.0001) for incident DM compared with the comparison cohort. The reduced diabetic risk in dialysis cohort was attributed to the lower diabetogenic effect of HD (adjusted HR 0.46, *p* value < 0.0001) rather than that of PD (adjusted HR 0.84, *p* value = 0.56). The significant associations were also consistently found when running stepwise Cox models. Regarding mortality, PD, and HD cohorts had significantly higher mortality risk in both Cox models.

### 4.3. Sensitivity Analysis

Table 3 showed the results of the four sensitivity analyses to corroborate the previous models.

In all assessments, a significant negative correlation between dialysis patients and DM events further confirmed the finding that dialysis is a protective factor in the development of DM. HD was significantly associated with a reduced risk of incident DM compared to the comparison cohort, while the risk between PD and comparison cohort did not differ in the four sensitivity analyses. Regarding non-DM death, dialysis cohort was associated with higher mortality risk than the comparison cohort.

### 4.4. Association of Dialysis with Incident DM Stratified by Sex, Age, Year of Enrollment, Numbers of Medical Visits, and Economic Incomes

Dialysis cohort was associated with a lower risk of incident DM than the comparison cohort in all the subgroup analyses, while a significantly higher mortality risk was seen in the dialysis cohort (Table 4).

### 4.5. Risk Factors for Incident DM among Dialysis Patients

We further determine the contributing factors to incident DM in the study population (Table 5). Among the entire study population, the factors contributing to the occurrence of incident DM consisted of hypertension, gout, and the user of statins with the presence of hypertension having the greatest risk being adjusted HR of 1.95 (95% CI 1.62–2.35, *p* value < 0.0001). The risk factors were not exactly the same among the whole study population, dialysis population and comparison population. In dialysis cohort, age was the only contributing factor for incident DM with an adjusted HR of 1.02 (1.01–1.04).

## 5. Discussion

To the best of our knowledge, our study is the first one using a representative nationwide data, to quantify the incidence rate of DM in patients undergoing dialysis compared with a non-dialysis reference cohort. Strikingly, ESRD patients were associated with a lower risk of incident DM (aHR 0.49; 95% CI 0.39–0.61, *p* < 0.0001), which was attributed to HD (aHR 0.46; 95% CI 0.37–0.58, *p* < 0.0001), not to PD (aHR 0.84, 95% CI 0.47–1.51, *p* = 0.56). The contributors to incident DM in the entire study cohort included hypertension, gout, and the use of statins.

Our findings were consolidated by the following reasons. First, we attempted to mitigate the impact of different patient’s characteristics distributions on the measured outcomes in the study cohorts by adjusting the propensity scores. Second, all established confounding factors for DM, including age, gender, comorbidities, and some pharmacotherapies were adjusted in competing- risk Cox models, and thus these covariates could not explain the reduced risk of incident DM in relation with dialysis. Third, the number of medical visits was also adjusted in the multivariate Cox models, so the detection bias, possibly caused by more frequent visits in dialysis patients, was minimized. Fourth, in our study, ESRD is, as expected, to associate to higher mortality risk, but surprisingly relate to lower incident DM risk when compared with non-ESRD. One may attribute those observations of our study to the higher death rates occurring in the ESRD patients, thus preventing them from developing DM. In order to resolve this issue, Fine and Gray method of competing risk analysis was chosen in our investigation, which more substantiated our findings.

The practice of dialysis procedures is very complex and various aspects related to dialysis per se and patients have different or even oppose effects on the pathogenesis of DM. Most of the available data suggested the liability of ESRD patients to develop DM. For example, intake of foods with high glycemic index is believed to predispose to postprandial hyperglycemia and higher insulin levels. The results from epidemiological studies on glycemic load in association with incident DM were contradictory. Villegas et al. reported that high intake of foods with a high glycemic index and glycemic load, especially rice, may increase the risk of type 2 DM in Chinese women, but this association was not found in the Whitehall II study [4,5]. Regardless, two meta-analyses, one of prospective cohort studies and the other of retrospective studies, reported a positive association of both dietary glycemic index and glycemic load and risk of type 2 DM [13,14]. The glucose content in the dialysate is another source of caloric supply in the dialysis patients. Based on our findings, the association between ESRD and incident DM could not be solely explained by glucose load.

Tremendous contributors to the development of DM have been identified with IR being one of the most important factors. IR exists in every stage of CKD and the etiologies are multifactorial, including vitamin D deficiency, erythropoietin deficiency, uremia milieu, inflammation, and hyperparathyroidism [15]. In addition, many comorbid factors, not related to CKD itself, contributing to IR, include old age, obesity, dyslipidemia, and hypertension, and so forth. IR has been shown to increase in ESRD patients, but dialysis treatment was reportedly capable of alleviating resistance. Using the euglycemia hyperinsulinaemic glucose clamp technique, Kabayashi et al. reported that both HD and PD can improve IR observed in uremia milieu [6]. Similar findings were also documented by Satirapoj et al. who reported decreased IR after five weeks of HD and PD in the same patient group [16]. However, no significant difference was found between predialysis and dialysis groups in a later investigation [17]. Therefore, once CKD patients do not develop DM before reaching ESRD, dialysis treatments may have positive effects on IR, thus reducing the incident DM risk.

Of the risk factors for type 2 DM, increasing body weight has been reported to one of the most important contributors to impaired glucose tolerance or even type 2 DM. Body mass index (BMI) was the greatest contributor among the three covariates (age, race/ethnicity, BMI) to the increase in diabetes prevalence after adjustments in a study of five NHANES involving 23,932 participants aged 20 to 74 years [18]. The nutritional parameters showed a longitudinal decline with dialysis vintage in dialysis patients. In an analysis of 17,022 patients commencing PD or HD, the BMI trajectory changed in a non-linear fashion, where mean BMI initially decreased, followed by increment and then stabilization at three years [19]. Afterwards, it dropped gradually. The protein-energy wasting (PEW) in ESRD patients on dialysis is caused by uremic toxins, inadequate dietary intake due to anorexia, and inflammation, and is closely associated with mortality [20]. Therefore, PEW or malnutrition may contribute to both lower incidence rate of DM and higher mortality rate. However, we did not have the BMI values for the present study.

Patients with DM and CKD have been found to require reduced or even discontinued of antidiabetic medication with the progression of CKD, initiation of dialysis therapy and gradual loss of residual renal function with time [21]. “Burnt-out diabetes” refers to the situation where diabetic patients can attain appropriate glycemic control, e.g., HbA1C < 6%, without any antidiabetic agents. A great number of mechanisms contributing to this phenomenon consist of loss of dietary intake due to diabetic gastroparesis and uremia, diminished renal and hepatic clearance of insulin with prolongation of insulin half-life, impaired renal gluconeogenesis, protein-energy wasting, disrupted counter-regulation of hypoglycemia, imposed dietary restriction, and hypoglycemia effects of dialysis [22,23]. Burnt-out diabetes was reported in 20.7% and 5.4% of Japanese diabetic patients undergoing HD by using glycated hemoglobin and glycated albumin, respectively [21]. Therefore, considering these effects underlying “burnt-out diabetes” on glucose homeostasis among non-diabetic patients at the initiation of dialysis, dialysis procedure per se might prevent those from developing DM.

In dialysis modalities (HD or PD), no consistent conclusion was drawn on which one is preferable to the other in terms of clinical outcomes. Typically, uremic toxins and excessive fluid have been removed intermittently in HD and continuously in PD. While higher inflammation was observed in oxidative stress and IR in ESRD, the influence of PD and HD on them were not exactly the same. Initiation of dialysis can improve IR and glucose tolerance. However, glucose load and absorption was also different. Glucose load is continuous in the PD patients throughout the day, whereas approximately 15–25 g of glucose may be removed during HD with the net absorption of glucose determined by the concentration of glucose-containing hemodialysates [24,25]. The glucose concentrations used to achieve appropriate ultrafiltration range from 1360 to 3860 md/dL and the glucose load delivered by PD can be as much as 10% to 30% of a patient’s total energy intake [26,27]. Burnt-out diabetes was only evidenced in HD, not in PD. Therefore, the effects of PD and HD on the occurrence of incident DM were different and the reduced risk of DM was only documented for HD.

CKD was shown to complicate IR, mainly due to post-receptor defect of insulin action, and may be prone to the development of incident DM in some reports. The aim of our study was to address whether the dialysis per se would increase or reduce the incidence of DM, so those pre-dialysis CKD patients developing DM in their CKD period ahead of ESRD requiring dialysis were excluded from our case cohort. We proposed that the remaining ESRD patients without developing DM in the CKD period may have some protective factors in the environmental and genetic aspects that mitigate the pro-diabetic tendency. Those protective factors may continue or even amplify once they progress to ESRD. This can partially explain why ESRD was associated with a lower risk of incident DM, although their influence cannot be comprehensively addressed in this study. 

As with other nationwide studies using Taiwan NHRID, there are several limitations in our study. First, some known contributing factors to incident DM, such as body mass index, physical activity, family history of DM, smoking, alcohol consumption, quantity and quality of sleep and dietary patterns, were not available in the NHRID. Those factors usually lead to cardiovascular comorbidities, which were controlled in our study, so we believe the bias caused by un-adjustment of those factors could be minimized. Second, four types of peritoneal dialysis solution (PDS) have been available in Taiwan: (1) The conventional glucose-based PDS; (2) neutral- pH PDS with low concentration of glucose degradation products; (3) icodextrin-based PDS; and (4) amino acid- containing PDS [28]. Many studies reported that biocompatible PDS led to less inflammation as compared to bio-incompatible PDS. Long-term online hemodiafiltration with ultrapure solution reportedly caused less inflammation via enhanced clearance of middle molecules than low-flux hemodialysis [29]. We cannot compare the influence of the four types of PDS and these two HD modes on glucose homeostasis due to technical limitations. Third, the number of patients on PD was relatively small, which may lead to the over- or underestimation of incident diabetic risk with PD. Therefore, the risk of diabetes in PD should be interpreted with caution. Fourth, the lack of laboratory data in our study is one limitation of NHRID. However, in order to avoid mis-coding problems, the use of DM’s ICD-9 code and anti-diabetic drugs is a prerequisite for diagnosing DM to improve diagnostic accuracy.

In conclusion, this study highlights the significant reduction in the risk of developing diabetes in patients undergoing dialysis and this association was attributed to HD rather than PD. In addition to Cox models of Fine and Gray’s method, stepwise cause-specific hazard models and sensitivity tests further corroborated this paradoxical relationship. Large-scale prospective studies on the underlying mechanisms of this phenomenon are urgent.

## Figures and Tables

**Table 1:** Baseline characteristics and clinical outcomes between the dialysis and comparison cohorts.

No. of Patients	Frequency Matched by Age, Gender, Index Year (1:4)		*p*-Value
	ESRD Dialysis Patient	Comparison Cohort	
	2228	8912	
**Demographics**			
Age	60.29 ± 16.43	60.29 ± 16.43	1.000
Gender, Male	1096 (49.19%)	4384 (49.19%)	1.000
The year of diagnosis			
2000	223 (10.01%)	892 (10.01%)	1.000
2001	122 (5.48%)	488 (5.48%)	1.000
2002	138 (6.19%)	552 (6.19%)	1.000
2003	139 (6.24%)	556 (6.24%)	1.000
2004	159 (7.14%)	636 (7.14%)	1.000
2005	168 (7.54%)	672 (7.54%)	1.000
2006	176 (7.9%)	704 (7.9%)	1.000
2007	159 (7.14%)	636 (7.14%)	1.000
2008	167 (7.5%)	668 (7.5%)	1.000
2009	157 (7.05%)	628 (7.05%)	1.000
2010	172 (7.72%)	688 (7.72%)	1.000
2011	162 (7.27%)	648 (7.27%)	1.000
2012	174 (7.81%)	696 (7.81%)	1.000
2013	112 (5.03%)	448 (5.03%)	1.000
Geographic location			
Northern Taiwan	958 (43%)	3846 (43.16%)	0.912
Central Taiwan	428 (19.21%)	1643 (18.44%)	0.418
Southern Taiwan	796 (35.73%)	3208 (36%)	0.832
Eastern Taiwan and islands	46 (2.06%)	215 (2.41%)	0.372
Monthly income, NTD	14,054.51 ± 13,083.05	14,383.13 ± 13,818.97	0.310
Number of medical visits in 1 year after study entry	35.62 ± 19.28	26.34 ± 18.42	<0.001
**Pre-existing comorbidities**			
Hypertension	1757 (78.86%)	6986 (78.39%)	0.628
Gout	473 (21.23%)	1337 (15%)	<0.001
Ischemic heart disease	400 (17.95%)	1373 (15.41%)	0.003
Congestive heart failure	430 (19.3%)	835 (9.37%)	<0.001
Cerebrovascular disease	181 (8.12%)	691 (7.75%)	0.561
Chronic obstructive pulmonary disease	297 (13.33%)	1051 (11.79%)	0.047
Rheumatoid disease	83 (3.73%)	280 (3.14%)	0.165
Charlson’s comorbidity index score	3.95 ± 1.84	2.55 ± 1.84	<0.001
**Long-term medication use**			
Anti-hypertensive drugs	1563 (70.15%)	5419 (60.81%)	<0.001
ACEIs/ARBs	1085 (48.7%)	3418 (38.35%)	<0.001
Diuretics	873 (39.18%)	2306 (25.88%)	<0.001
Beta-blockers	946 (42.46%)	3219 (36.12%)	<0.0001
NSAIDs	231 (10.37%)	737 (8.27%)	0.002
Analgesic drugs other than NSAIDs	320 (14.36%)	875 (9.82%)	<0.001
Statin	437 (19.61%)	1186 (13.31%)	<0.001
Corticosteroid	245 (11%)	499 (5.6%)	<0.001
Outcome			
New-onset DM	109 (4.89%)	869 (9.75%)	<0.001
Death	671 (30.12%)	1250 (14.03%)	<0.001
Follow-up time (years)	5.35 ± 3.92	6.14 ± 3.95	<0.001

Values are expressed as mean ± SD or number (percentage). Abbreviations: ACE inhibitor, angiotensin-converting enzyme inhibitor; ARB, angiotensin II receptor blocker; NSAID, Non-Steroidal Anti-Inflammatory Drug; NTD, New Taiwan Dollar.

**Table 2:** Incidence and risk of new-onset DM and mortality between ESRD dialysis cohort and the comparison cohort.

	Events (n/N)	Incidence Rate ^a^	cHR (95% CI)	*p* Value	aHR ^b^ (95% CI)	*p* Value	aHR ^c^ (95% CI)	*p* Value
**New-onset DM**								
Cohorts								
Comparison cohort	869/8912	15.88 (14.83–16.94)	1.00	—	1.00	—	1.00	—
ESRD dialysis cohort	109/2228	9.15 (7.43–10.87)	0.50 (0.41–0.61)	<0.0001	0.49 (0.39–0.61)	<0.0001	0.56 (0.46–0.69)	<0.0001
*Categorized dialysis type at start*								
Comparison cohort	869/8912	15.88 (14.83–16.94)	1.00	—	1.00	—	1.00	—
ESRD dialysis cohort								
PD	12/136	15.98 (6.94–25.03)	0.94 (0.53–1.66)	0.82	0.84 (0.47–1.51)	0.56	0.93 (0.52–1.64)	0.8
HD	97/2092	8.69 (6.96–10.42)	0.47 (0.38–0.58)	<0.0001	0.46 (0.37–0.58)	<0.0001	0.54 (0.44–0.66)	<0.0001
**Mortality**								
Cohorts								
Comparison cohort	1250/8912	22.85 (21.58–24.11)	1.00	—	1.00	—	1.00	—
ESRD dialysis cohort	671/2228	56.33 (52.06–60.59)	2.54 (2.31–2.79)	<0.0001	2.32 (2.07–2.60)	<0.0001	2.25 (2.00–2.47)	<0.0001
*Categorized dialysis type at start*								
Comparison cohort	1250/8912	22.85 (21.58–24.11)	1.00	—	1.00	—	1.00	—
ESRD dialysis cohort								
PD	29/136	38.62 (24.57–52.68)	1.67 (1.16–2.40)	0.006	2.37 (1.61–3.51)	<0.0001	2.50 (1.72–3.63)	<0.0001
HD	642/2092	57.52 (53.07–61.97)	2.60 (2.36–2.86)	<0.0001	2.32 (2.07–2.60)	<0.0001	2.21 (1.99–2.46)	<0.0001

Abbreviations: ESRD, end-stage renal disease; aHR, adjusted hazard ratio; cHR, crude hazard ratio; CI, confidence interval. ^a^ per 1000 person-years. ^b^ Adjusted for all variables in Table 1 by Cox proportional hazard model with Fine and Grey’s method to consider death as a competing risk. ^c^ Adjusted for all variables in Table 1 by stepwise Cox proportional hazard model (SLENTRY = 0.15 and SLSTAY = 0.15) with cause-specified method to consider death as a competing risk.

**Table 3:** Sensitivity analysis.

	New-Onset DM				Non-DM Death			
	aHR ^a^ (95% CI)	*p* Value	aHR ^b^ (95% CI)	*p* Value	aHR ^a^ (95% CI)	*p* Value	aHR ^b^ (95% CI)	*p* Value
**Adjusted for the propensity score**								
Comparison cohort	1.00	—	1.00	—	1.00	—	1.00	—
ESRD dialysis cohort	0.53 (0.43–0.65)	<0.0001	0.57 (0.46–0.7)	<0.0001	1.85 (1.66–2.06)	<0.0001	1.78 (1.6–1.98)	<0.0001
PD	0.98 (0.55–1.74)	0.94	0.99 (0.56–1.75)	0.96	1.89 (1.7–2.11)	<0.0001	1.81 (1.63–2.01)	<0.0001
HD	0.50 (0.40–0.63)	<0.0001	0.54 (0.43–0.67)	<0.0001	1.24 (0.85–1.81)	0.27	1.29 (0.89–1.86)	0.18
**Kidney transplant as a censored covariate**								
Comparison cohort	1.00	—	1.00	—	1.00	—	1.00	—
ESRD dialysis cohort	0.50 (0.40–0.63)	<0.0001	0.53 (0.43–0.65)	<0.0001	2.32 (2.07–2.59)	<0.0001	2.24 (2.02–2.49)	<0.0001
PD	0.87 (0.46–1.64)	0.66	0.89 (0.47–1.65)	0.7	2.30 (1.54–3.44)	<0.0001	2.46 (1.67–3.61)	<0.0001
HD	0.47 (0.37–0.60)	<0.0001	0.50 (0.40–0.63)	<0.0001	2.31 (2.06–2.58)	<0.0001	2.22 (1.99–2.47)	<0.0001
**Change of renal replacement therapy as time-dependent covariates**								
Comparison cohort	1.00	—	1.00	—	1.00	—	1.00	—
ESRD dialysis cohort	0.54 (0.43–0.67)	<0.0001	0.53 (0.43–0.66)	<0.0001	2.31 (2.06–2.58)	<0.0001	2.23 (2–2.47)	<0.0001
PD	0.96 (0.54–1.71)	0.88	0.93 (0.51–1.69)	0.81	2.49 (1.65–3.77)	<0.0001	2.47 (1.63–3.75)	<0.0001
HD	0.51 (0.40–0.64)	<0.0001	0.50 (0.40–0.62)	<0.0001	2.30 (2.06–2.57)	<0.0001	2.22 (1.99–2.47)	<0.0001
**Change of dialysis mode or kidney transplant as censored covariates**								
Comparison cohort	1.00	—	1.00	—	1.00	—	1.00	—
ESRD dialysis cohort	0.57 (0.45–0.71)	<0.0001	0.61 (0.5–0.75)	<0.0001	2.43 (2.18–2.72)	<0.0001	2.36 (2.13–2.62)	<0.0001
PD	1.13 (0.6–2.14)	0.7	1.37 (0.73–2.56)	0.33	3.8 (2.5–5.79)	<0.0001	4.39 (3.02–6.38)	<0.0001
HD	0.54 (0.43–0.68)	<0.0001	0.59 (0.47–0.73)	<0.0001	2.4 (2.14–2.68)	<0.0001	2.32 (2.08–2.57)	<0.0001

Abbreviations: aHR, adjusted hazard ratio; CI, confidence interval; HD, hemodialysis; PD, peritoneal dialysis. ^a^ Adjusted for all variables in Table 1 by Cox proportional hazard model with Fine and Grey’s method to consider death as a competing risk. ^b^ Adjusted for all variables in Table 1 by stepwise Cox proportional hazard model (SLENTRY = 0.15 and SLSTAY = 0.15) with cause-specified method to consider death as a competing risk.

**Table 4:** Subgroup analyses of risk for DM and mortality between the ESRD and control cohorts. ^a.^

Subgroup		Comparison Subjects			ESRD Subjects			ESRD Subjects vs. Comparison Subjects					
								Risk of DM			Risk of Non-DM Death		
		*N*	DM	Non-DM Death	*N*	DM	Non-DM Death	aHR (95% CI)	*p* Value	P_interaction_	aHR (95% CI)	*p* Value	P_interaction_
Gender													
	Female	4528	470	531	1132	61	302	0.48 (0.36–0.64)	<0.001	0.76	2.44 (2.05–2.91)	<0.0001	0.86
	Male	4384	399	719	1096	48	369	0.50 (0.36–0.71)	<0.001		2.25 (1.94–2.62)	<0.0001	
Age, years													
	<65	4968	563	371	1242	65	223	0.43 (0.33–0.58)	<0.001	0.28	1.82 (1.49–2.23)	<0.001	0.76
	≥65	3944	306	879	986	44	448	0.63 (0.44–0.89)	0.010		2.40 (2.09–2.76)	<0.001	
Year of index													
	2000–2005	3796	573	695	949	82	340	0.61 (0.47–0.79)	<0.001	0.03	2.03 (1.73–2.39)	<0.0001	<0.001
	2006–2013	5116	296	555	1279	27	331	0.39 (0.26–0.60)	<0.001		2.79 (2.37–3.28)	<0.0001	
Income (New Taiwan dollars)													
	<15,840	4740	412	852	1177	52	403	0.59 (0.43–0.82)	0.001	0.62	2.18 (1.89–2.51)	<0.001	0.04
	≥15,840	4172	457	398	1051	57	268	0.49 (0.36–0.66)	<0.001		2.58 (2.13–3.13)	<0.001	
Number of medical visits in 1 year after study entry													
	<24	4812	471	587	643	24	213	0.39 (0.25–0.60)	<0.001	0.26	4.09 (3.39–4.94)	<0.001	<0.001
	≥24	4100	398	663	1585	85	458	0.59 (0.46–0.76)	<0.001		1.83 (1.59–2.09)	<0.001	

Abbreviations: aHR, adjusted hazard ratio; CI, confidence interval; ^a^ Adjusted for all variables in Table 1.

**Table 5:** The risk factors associated with of new onset DM ^a^.

Risk Factor	Study Patients (*N* = 11140)		Comparison Cohort (*N* = 8912)		ESRD Dialysis Cohort (*N* = 2228)	
	aHR ^b^ (95% CI)	*p* Value	aHR ^b^ (95% CI)	*p* Value	aHR ^b^ (95% CI)	*p* Value
ESRD dialysis	0.56 (0.46–0.69)	<0.0001	—	—	—	—
Age	—	—	—	—	1.02 (1.01–1.04)	0.0009
Gender, Male	0.9 (0.79–1.02)	0.11	—	—	—	—
Geographic location						
Northern Taiwan	—	—	1 (reference)		—	—
Central Taiwan	—	—	1.15 (0.95–1.39)	0.14	—	—
Southern Taiwan	—	—	1.23 (1.05–1.43)	0.008	—	—
Eastern Taiwan and islands	—	—	1.02 (0.65–1.61)	0.92	—	—
Monthly income	—	—	—	—	1.13 (0.98–1.29)	0.09
Hypertension	1.95 (1.62–2.35)	<0.0001	2.12 (1.74–2.59)	<0.0001		
Gout	1.36 (1.15–1.6)	0.0002	1.32 (1.11–1.56)	0.0015	1.44 (0.93–2.23)	0.1
Analgesic drugs other than NSAIDs	0.81 (0.63–1.04)	0.09	0.78 (0.6–1.03)	0.08	—	—
statin	1.27 (1.06–1.52)	0.009	1.34 (1.11–1.62)	0.0026	—	—
Anti-hypertensive drugs	—	—	—	—	0.71 (0.47–1.06)	0.09

Abbreviations: aHR, adjusted hazard ratio; CI, confidence interval. ^a^ only the variables with a *p* value < 0.15 were listed. ^b^ Adjusted for all variables in Table 1 by Cox proportional hazard model with Fine and Grey’s method to consider death as a competing risk.
